# *Notes from the Field:* Verona Integron-Encoded Metallo-Beta-Lactamase–Producing *Pseudomonas aeruginosa* Outbreak in a Long-Term Acute Care Hospital — Orange County, Florida, 2017

**DOI:** 10.15585/mmwr.mm6721a6

**Published:** 2018-06-01

**Authors:** Danielle Rankin, Luz Caicedo, Nychie Dotson, Paige Gable, Alvina Chu

**Affiliations:** ^1^Florida Department of Health in Orange County, Florida; ^2^Florida Department of Health Bureau of Epidemiology; ^3^Division of Healthcare Quality Promotion, Office of Infectious Diseases, CDC.

On July 5, 2017, one case of colonization with Verona integron-encoded metallo-beta-lactamase (VIM)–producing *Pseudomonas aeruginosa* was identified at a long-term acute care hospital (LTACH) in Orange County, Florida. VIM genes are capable of transferring among bacterial species (*1*); however, the mechanisms and frequency of resistance exchange is poorly understood (*2*). Thus, identification of colonization with VIM-resistant organisms is a sentinel event that warrants investigation and careful patient management. In response, the patient was placed on contact precautions and a facilitywide point prevalence survey was conducted (*3*). To detect colonization of VIM-producing *P. aeruginosa,* rectal swabs were collected from patients at the LTACH. The Florida Department of Health collaborated with the Tennessee Department of Health, the Southeast Regional Antibiotic Resistance Laboratory Network in Tennesee, and CDC to conduct antimicrobial resistance testing and genotyping.

During July 13–September 22, 2017, six additional patients at the LTACH screened positive for VIM-producing *P. aeruginosa* during three biweekly point prevalence surveys and an enhanced prospective surveillance system (Figure). The median length of stay at the LTACH among the seven colonized patients was 40.5 days (range = 13–150 days), and their median age was 60 years (range = 40–68 years); 57% were men. No patients reported hospitalizations or medical procedures outside the United States. Among the seven colonized patients, six had tracheostomy tubes (including three with current diagnoses of ventilator-dependent respiratory failure), six had decubitus ulcers, and four were receiving hemodialysis. Five patients had received antibiotic therapy before specimen collection, and one patient died approximately 1 month after colonization was detected. No cases of infection or complications associated with VIM-producing *P. aeruginosa* colonization have been reported at the LTACH. Pulsed field gel electrophoresis was conducted on four of the seven isolates; two had closely related (>90% similarity) patterns. 

**FIGURE:**
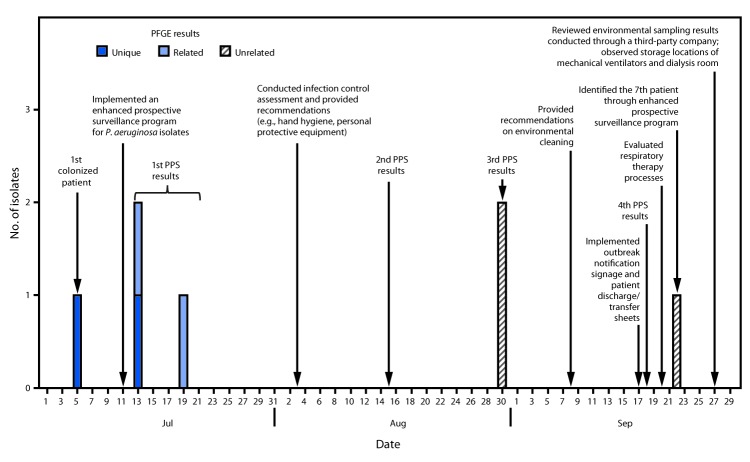
Colonization of patients at a long-term acute care hospital with Verona integron-encoded metallo-beta-lactamase–producing *Pseudomonas aeruginosa*, timing of point prevalence surveys and implementation of infection control, and isolate pulsed-field gel electrophoresis (PFGE) results — Orange County, Florida, July–September, 2017 **Abbreviation:** PPS = point prevalence survey.

This investigation documents the first identification of VIM-producing *P. aeruginosa* in Florida. VIM-producing *P. aeruginosa* was first reported in Marseilles, France, in 1996 and has since been documented in health care–associated infections in several countries (*4*,*5*). Transmission can occur horizontally via hand carriage by health care personnel, through shared medical equipment, and through fomites (e.g., bedside tables, intravenous poles, bedside commodes, and sink drains) (*4*). Control measures include enhancing and reinforcing infection control processes and environmental disinfection. Measures taken in response to this outbreak investigation include 1) implementing an enhanced prospective surveillance program for *P. aeruginosa* isolates, 2) conducting infection control and response assessments (e.g., hand hygiene, personal protective equipment), 3) observing and reinforcing environmental cleaning practices, 4) implementing outbreak notification signage and patient discharge/transfer sheets, and 5) evaluating respiratory therapy processes.

Although carbapenem-resistant *P. aeruginosa* can be identified through routine culture and susceptibility testing, testing for mechanisms of resistance are not readily accessible. To detect the VIM-producing gene, additional antimicrobial resistance mechanism testing by polymerase chain reaction (PCR) must be conducted. Such testing is not routinely conducted at most clinical laboratories but is available now in all 50 states via CDC’s Antimicrobial Resistance Laboratory Network (ARLN).

Routine surveillance or PCR testing for antibiotic resistance mechanisms among *P. aeruginosa* is not practiced widely or uniformly; thus, the true incidence and prevalence of VIM-producing *P. aeruginosa* in the community and the risk for transmission among patients in health care facilities is unknown (*6*). Testing for common carbapenemases via the ARLN has the potential to better define the epidemiology of carbapenem-resistant *P. aeruginosa* resistance mechanisms as well as inform the response to control transmission. Reporting of organisms with high-priority antibiotic resistance mechanisms to public health authorities can inform regional infection control and containment practices.
